# Software-Enhanced Drugs™: The third platform reshaping drug development

**DOI:** 10.1073/pnas.2522286122

**Published:** 2025-09-19

**Authors:** Shaheen E. Lakhan, Paul Wicks

**Affiliations:** ^a^Click Therapeutics, Inc., New York, NY 10013; ^b^Wicks Digital Health Ltd., Lichfield WS13 6DN, United Kingdom

Jones et al. (1) describe two life sciences platforms (molecule-based and design-enabled) that create outsized strategic value and accelerate clinical development. Using their Platform Value Identification across Strategic, Technical, and Adaptive domains (VISTA) framework, they describe the biotech case studies of Alnylam’s RNAi therapeutics platform, Genentech’s therapeutic antibody platform, and Moderna’s mRNA platform. We believe the recent FDA draft guidance document “Regulatory Considerations for Prescription Drug Use-Related Software” (PDURS) (2) enables a new platform, the combination of drugs with prescription digital therapeutics to form new “Software-Enhanced Drugs.”

Software-enhanced drug treatments leverage validated mechanisms of action like cognitive behavioral therapy, psychoeducation, and neuromodulatory functions to offer software-based drivers of efficacy that adjunctively enhance pharmacological effects. They also offer digital tools to enhance drug action via improving drug delivery, adherence, or drug-related clinical decision-making (3). While translating psychotherapeutic interventions into other media such as self-help books, education courses, or standalone computer programs is not new (4), the sharpening of regulatory definitions like “software as a medical device” (SaMD) (5) have required DTx developers to implement stringent quality management systems and conduct robust evidence generation studies akin to the pivotal trials of a new molecular entity (5). Examples include the recent FDA first-in-class marketing authorization of CT-132 for prevention of episodic migraine (6). The development of multiple DTx on a shared platform aligns with Jones et al.’s “design-enabled platforms” which inherently rely upon algorithms, data processing, and personalization to individualize therapy, monitor patient response, and provide just-in-time behavioral interventions.

A value-adding software-enhanced drug platform accelerates discovery by offering a database of translated clinically validated mechanisms of action from the behavioral health literature in digital therapeutic format, a process that can be augmented by AI. Reductions in redundancy can be achieved by applying consistent design principles to encourage patient engagement such as motivational interfaces, personalization, and therapy-specific tailoring through user-centered design. Innovations in these offerings can be introduced at a rate unencumbered by the safety concerns of traditional drug development. The prior experience of adjacent therapeutic areas enhances the probability of success for new products. We have also seen the intangible value of a platform-based approach in attracting a dense talent pool of digital therapeutic specialists across psychology, design, mobile app development, data science, and commercialization; a process aided by collaboration with clinical partners looking to take their mechanisms of action into clinical practice via such successful platforms.

Just as biochemistry and biophysics platform technologies have transformed biopharma R&D, robust, clinically validated software-enhanced drug platforms have the potential to redefine therapeutic innovation in a matter of years not decades. While relatively early to assess core VISTA metrics, we will be watching the leading indicators closely. Recognizing the application of new approaches through frameworks like VISTA is crucial for fostering investment, accelerating innovation, and ultimately delivering more effective, personalized, and adaptive healthcare solutions. It is our shared hope that this holistic understanding will catalyze the development of next-generation therapeutics.
